# Effects of Storage Temperature and Packaging Type on the Postharvest Quality of Finger Lime (*Citrus australasica*)

**DOI:** 10.3390/foods15193476

**Published:** 2026-09-28

**Authors:** Hanieh Noeparast, Sunil K. Panchal, Paul Holford, Michelle Donovan-Mak

**Affiliations:** School of Science, Faculty of Engineering, Computing and Science, Western Sydney University, Hawkesbury Campus, Richmond, NSW 2753, Australia; 22113433@student.westernsydney.edu.au (H.N.); s.panchal@westernsydney.edu.au (S.K.P.); p.holford@westernsydney.edu.au (P.H.)

**Keywords:** *Citrus australasica*, postharvest quality, packaging, storage temperature, phenolic content, flavonoid content, antioxidant capacity, chilling injury

## Abstract

Finger lime (*Citrus australasica* F.Muell.) is a native Australian citrus species with increasing commercial value because of its unique sensory, visual and nutritional properties. However, like other citrus species, it is highly perishable and susceptible to postharvest quality deterioration. Despite its growing importance, limited research has examined how commercial packaging and storage temperature affect its quality. Therefore, this study investigated the combined effects of three commonly used types of packaging (net bags, recycled polyethylene terephthalate (rPET) punnets and moulded cardboard clamshells) and three storage temperatures (room temperature (RT; ~25 °C), 8 °C and 4 °C) on finger lime quality over eight weeks. The quality parameters examined included weight loss, firmness, colour, visual appearance and overall marketability, as well as nutritional properties, including total phenolic content, total flavonoid content and antioxidant capacity. Packaging and temperature both significantly affected fruit marketability. Fruit stored at RT quickly lost quality in all packaging types. Fruit stored at 4 °C retained firmness, colour, total phenolic content, flavonoid content and antioxidant capacity better than fruit stored at 25 °C or 8 °C; however, chilling injury and fungal growth developed at 4 °C, with approximately 20% of the fruit in each packaging treatment deemed unsaleable by Week 8. At 8 °C, no chilling injury, visible fungal growth or shrivelling was observed. Although approximately 60% of fruit in cardboard clamshells and net bags were classified as unsaleable because of loss of freshness, approximately 80% of fruit in punnets remained saleable until the end of the trial. Overall, storage at 8 °C in rPET punnets provided the best balance between quality retention and marketability among the combinations of packaging type and storage temperature examined in this study.

## 1. Introduction

Australia is home to a unique diversity of native citrus, encompassing seven recognised species [1,2]. In addition to contributing to Australia’s native biodiversity, these species are an integral part of Australia’s growing ‘bushfood’ industry. The best-known and most commercially utilised of these species is the finger lime (*Citrus australasica* F.Muell.). The market value of this crop is rising in both domestic and international markets owing to its high demand and global appeal in gourmet and specialty food markets [3]. Owing to its evolution in isolation from Asia, finger lime has developed a distinctive morphology, producing ovoid fruit that, as the name suggests, resembles a finger in shape and size. The pulp consists of hundreds of small, spherical juice vesicles that can be extruded to produce finger lime ‘caviar’. When eaten, these vesicles burst in the mouth and release juice with a distinctive nutrient and sensory profile, contributing to the fruit’s culinary appeal in high-end restaurants in the Asia-Pacific, Europe and North America, where it is used as a flavour enhancer in cocktails, crackers, drinks and salads [4]. In addition, the albedo is small compared with that of other citrus species [5], resulting in a high pulp-to-fruit ratio. Finger lime is a genetically diverse species; consequently, its fruit varies in size, shape, skin hue and texture, and seed number [6], with skin colours ranging from green to red, brown or purple and pulp colours ranging from green to yellow, red or pink [7]. This diversity has supported its use in breeding programs for many years, and more than 30 varieties are now grown by Australian producers [3].

Citrus species are among the most important fruit crops [8,9]. In addition to contributing to dietary requirements through their high contents of vitamins, minerals and fibre, citrus fruits have applications in the pharmaceutical [10] and cosmetic industries [11] because of their high concentrations of bioactive compounds. Citrus fruits contain substantial concentrations of flavonoids, including flavanone-7-O-glycosides, flavones, polymethoxylated flavones, flavonols and anthocyanins [12,13,14,15]. Several of these compounds have been shown to provide health benefits, primarily through their antioxidant, anti-inflammatory and lipid-lowering properties [16,17]. Finger limes are no exception and are rich in minerals, carbohydrates and bioactive compounds such as phenolic acids, flavanols and flavonols [4,5,18,19]. However, inappropriate postharvest storage conditions can reduce the concentrations of bioactive compounds, thereby decreasing the nutritional value of the fruit for consumers [20,21,22].

Fresh horticultural produce remains metabolically active after harvest and progressively deteriorates because of respiration, transpiration, senescence and microbial activity [23,24]. In citrus fruit, postharvest deterioration is caused by continued respiration and transpiration, water loss, tissue senescence, peel injury, physiological disorders and fungal infection. Inappropriate temperature and relative humidity further accelerate quality loss [21,25]. Finger lime fruit, like other citrus species, can be highly perishable and susceptible to postharvest quality loss and, therefore, requires careful handling, storage and packaging to preserve quality and minimise losses [26,27]. Postharvest diseases caused by pathogens, as well as physiological disorders and peel injuries or bruising caused by improper handling, can directly or indirectly result in postharvest losses and food waste by reducing nutritional value and visual appeal or by causing spoilage and dehydration [25,28,29]. Storage temperature is an important factor affecting the postharvest quality of citrus fruit because it regulates physiological processes such as respiration, moisture loss and senescence [30]. Therefore, temperature management is one of the most effective approaches for slowing postharvest deterioration [23].

Although lower temperatures are commonly used for many commodities to reduce postharvest losses, finger limes are sensitive to chilling injury and may develop physiological disorders at low temperatures [6,23]. High storage temperatures, on the other hand, can accelerate fruit deterioration, weight loss and discolouration, resulting in reduced shelf life and marketability [31]. Commodity-specific packaging provides physical protection during storage and transportation and may regulate gas exchange and moisture loss, thereby playing a critical role in reducing postharvest waste [32,33]. To date, there has been little evaluation of the combined influence of packaging and storage temperature on the postharvest quality of finger lime, particularly with respect to bioactive compounds. Therefore, this study aimed to investigate the effects of three commercial packaging types and three storage temperatures and to identify the most favourable conditions for the transport and storage of finger lime.

## 2. Materials and Methods

### 2.1. Source of Fruit, Packaging and Storage Conditions

Finger lime fruits (*Citrus australasica* F.Muell.) of the cultivar ‘Red Champagne’ were commercially harvested on 25 August 2024 from Lilyshire Finger Limes, Cardwell, QLD, Australia, and received at Western Sydney University on 29 August 2024. Prior to delivery, the fruits were washed by the grower in a 1% sodium hypochlorite solution for 1–2 min as part of their routine postharvest treatment. Fruits with visible mechanical damage, bruising, cracking, decay, or marked deformity were excluded prior to packaging. Biological variation was minimised by using a single cultivar from the same commercial source and randomly allocating the selected fruit among treatments. Three types of packaging were used: (1) net bags on an open tray, to simulate the open packaging commonly used for domestic retail purposes; (2) perforated 125 g recycled polyethylene terephthalate (rPET) blueberry punnets, to determine the feasibility of repurposing an existing retail-format for a cross-commodity application; and (3) moulded cardboard Eco Clamshell boxes containing a transparent window, representing an environmentally friendly, 100% recyclable and biodegradable option (Figure 1). These packaging types were evaluated for their effectiveness in retaining the physical attributes and nutritional properties of finger limes during postharvest storage. The punnets were purchased from Multistep Pty Ltd. (M-125C1, Silverwater, NSW, Australia), while the cardboard Eco Clamshell boxes were purchased from Roocreate (North Wollongong, NSW, Australia). Fruit stored in net bags constituted the control group. To simulate storage and transportation environments, three storage conditions were selected: (1) room temperature (RT), using a laboratory maintained at 25 ± 2 °C and 90 ± 5% relative humidity (RH) (ambient indoor temperature in Australia during the cropping season); (2) 8 °C in a cool room, selected as an intermediate refrigerated temperature within the reported 5–10 °C storage range for finger lime and above temperatures associated with increased chilling-injury risk; and (3) 4 °C in a cool room to simulate refrigerated transport [34,35,36,37,38,39]. At each temperature, there were three replicate packages of each packaging type, and each package contained five numbered fruits; one fruit from each replicate package was assessed at each sampling time, giving *n* = 3.

### 2.2. Assessment of Morphophysiological Attributes and Shelf Life

Morphophysiological attributes and nutritional properties of the fruit in each packaging × temperature combination were evaluated over a period of eight weeks, with measurements made at weekly or fortnightly intervals depending on the parameter assessed. Fruit weight loss was calculated using the following formula [40]:

Weight loss (%) = [(initial fruit weight − final fruit weight)/initial fruit weight] × 100

Firmness was determined using a texture analyser (TA-XT Plus, Stable Micro Systems Ltd., Godalming, Surrey, UK). Measurements were performed fortnightly using a puncture test with a 5 mm cylindrical probe. Each fruit was positioned horizontally on the platform, and the probe was driven through the intact peel and into the flesh to a penetration depth of 10 mm. The peak force required to penetrate the fruit to a depth of 10 mm was recorded as total force (g). The test parameters were standardised as follows: pre-test speed, 2 mm/s; test speed, 1 mm/s; and post-test speed, 2 mm/s [41].

Changes in finger lime colour were quantitatively measured using a chromameter (CR-410, Minolta, Tokyo, Japan) at four evenly distributed equatorial sites on each fruit. The L*, a* and b* colour values were recorded, and ΔE* values were calculated according to the method described by the Commission Internationale de l’Éclairage [42]. The chromameter was calibrated using the standard calibration plate provided by the manufacturer before each measurement session [43].

Fruit appearance was assessed visually on a weekly basis. Symptoms of chilling injury, fungal growth, shrivelling caused by moisture loss, and changes in peel colour were recorded during the eight-week storage period. Peel pitting, sunken brown lesions and scald-like discolouration were considered symptoms of chilling injury. The severity of chilling injury was evaluated using a four-point scale [44] for the visual assessment of citrus fruit, where 0 represents the absence of symptoms, 1 indicates slight pitting or browning, 2 indicates moderate pitting and tissue collapse, and 3 indicates extensive pitting that renders the fruit unsaleable [40,44,45,46,47]. Loss of freshness was defined as a decline in the overall visual appeal of the fruit despite the absence of visible symptoms of fungal growth, chilling injury or shrivelling.

Visible fungal growth was recorded separately and identified by the presence of mycelium, sporulation, soft or water-soaked lesions, or spreading tissue breakdown. Uniform changes in peel colour without associated pitting or tissue collapse were considered general colour changes rather than chilling injury [48]. The earliest appearance of chilling injury, fungal growth and shrivelling was recorded as the onset week. Fruits were classified as saleable when fungal growth, peel disorders, shrivelling and loss of visual freshness were absent or minimal, and as unsaleable when any of these attributes reduced the commercial acceptability of the fruit. The percentage of unsaleable fruit was calculated as the number of fruits classified as unsaleable divided by the total number of fruits assessed for each treatment, multiplied by 100.

### 2.3. Evaluation of Nutritional Properties

Fruit peel and pulp were separated, and 5 g of pulp was ground thoroughly using a pestle and mortar. The resulting slurry was mixed with 15 mL of a solution containing 80% aqueous methanol and 0.5 M hydrochloric acid (1:1, *v*/*v*) and incubated overnight at 4 °C. The mixture was centrifuged at 20,000 × *g* for 20 min at 4 °C, and the supernatant was collected for analysis of total phenolic content, flavonoid content and antioxidant capacity.

The Folin–Ciocalteu method was used to determine total phenolic content [49,50,51]. Initially, 0.5 mL of the supernatant was mixed with 8 mL of deionised water. Then, 0.5 mL of Folin–Ciocalteu reagent (Sigma-Aldrich, Bayswater, VIC, Australia) was added to the mixture. Subsequently, 1 mL of 20% (*w*/*v*) sodium carbonate solution was added; the solution was mixed for approximately 10 s and incubated for 1 h at 25 °C in the dark to allow reduction of the Folin–Ciocalteu reagent. Aliquots (200 µL) of the mixture were transferred to a 96-well microplate (Sarstedt, Nümbrecht, Germany), and absorbance was measured at 750 nm using a spectrophotometer (SpectroStar Nano, BMG Labtech, Mornington, VIC, Australia). The plate was shaken for 10 s before measurements were taken using the following parameters: beam length, 2 mm (with path-length correction), and flash count, 10. Results were expressed as gallic acid equivalents (mg gallic acid equivalent/100 g fresh weight) [19,49]. Gallic acid (Sigma-Aldrich) at concentrations of 0.025, 0.05, 0.075, 0.1, 0.15, 0.2, 0.25, 0.3, 0.35 and 0.4 mg/mL was used as the standard for determining total phenolic content.

A colourimetric assay was conducted to measure flavonoid content. An aliquot of 1 mL of supernatant was combined with distilled water in a 1:2 ratio before 0.3 mL of 5% sodium nitrite was added. Following a 5 min incubation, 0.3 mL of 10% aluminium chloride was added, forming a yellow complex. After 1 min, 2 mL of 1 M sodium hydroxide was added, and the mixture was vortexed, resulting in a reddish-brown solution. Absorbance was then measured at 510 nm, and the results were reported as catechin equivalents (mg catechin equivalent/100 g fresh weight) [19,52]. Catechin (Sigma-Aldrich) at concentrations of 0.025, 0.05, 0.075, 0.1, 0.15, 0.2, 0.25, 0.3, 0.35 and 0.4 mg/mL was used as the standard for determining total flavonoid content.

The ferric reducing antioxidant power (FRAP) method was used to determine antioxidant capacity. First, the FRAP reagent was prepared by mixing 2,4,6-tripyridyl-s-triazine solution (10 mmol/L) in 0.04 mol/L hydrochloric acid, freshly prepared 0.02 mol/L ferric chloride and 0.3 mol/L sodium acetate in a ratio of 1:1:10. The pH was adjusted to 3.6 using acetic acid. The mixture was then placed in a water bath at 37 °C for 30 min. After heating, 225 μL of the reagent was mixed with 22.5 μL of deionised water and 7.5 μL of the test supernatant. The mixture was incubated at 37 °C for a further 30 min. Finally, absorbance was measured at 595 nm using a spectrophotometer (SpectroStar Nano). Results were expressed as ferrous equivalents [Fe^2+^ eq (mmol/g fresh weight)] [19,53]. Ferrous sulphate at concentrations of 0.025, 0.05, 0.075, 0.1, 0.15, 0.2, 0.25, 0.3, 0.35 and 0.4 mg/mL was used to plot standard curves.

### 2.4. Statistical Analysis

Data sets were subjected to analysis of variance using Statistica (V14, TIBCO Software Inc., Palo Alto, CA, USA). ANOVA was performed independently at each assessment time point. Percentage data were subjected to an arcsine transformation before analysis. Means were separated using Fisher’s least significant difference (LSD) test at *p* = 0.05. The results of the analyses are presented in Supplementary Tables S1–S6.

## 3. Results and Discussion

ANOVA showed significant effects of storage temperature and packaging on the quantitative quality attributes of finger lime, with both main effects being highly significant (*p* < 0.001) for most variables and assessment times (Tables S1–S6). Significant temperature × packaging interactions were observed for weight loss at all assessment times, firmness at Weeks 4 and 8, flavonoid content at Weeks 2–6, and antioxidant capacity at Week 8 (*p* < 0.01).

### 3.1. Fruit Weight Loss

Water loss from horticultural produce is a major cause of postharvest deterioration and occurs through transpiration from the stem scar, stomata and cuticle. It causes reductions in fresh weight, resulting in economic losses and reductions in quality through visual, compositional and textural changes. Relatively small moisture losses are sufficient to cause shrivelling, wilting and undesirable texture changes [54]. In our study, progressive weight loss was observed over the eight-week storage period. At all assessment times, finger limes stored at 4 °C exhibited the least weight loss across all packaging types, whereas the highest weight loss was observed at RT (Figure 2A–C). These results are consistent with previous studies showing that higher temperatures increase transpiration rates and consequently increase weight loss [30,55,56,57,58]. However, the magnitude of weight loss varies among studies because transpiration is influenced by fruit characteristics including surface-area-to-volume ratio and peel properties, as well as storage temperature and relative humidity [57,59]. At all storage temperatures, fruit in rPET punnets showed the lowest weight loss, whereas the highest losses were observed in fruit stored in net bags. This may be associated with reduced moisture exchange between the fruit and the surrounding environment. In contrast, fruit in net bags were continuously exposed to moving air, which removes transpired water and maintains higher vapour pressure deficits. In addition to transpiration, weight loss also occurs through respiration, although losses from this process are generally considered minor except under conditions of high relative humidity [59].

### 3.2. Firmness

Fruit firmness is an important quality criterion for consumers [60], and fruit with greater moisture retention is often reported to have greater rind firmness and tissue turgidity [61,62]. Under all storage conditions, firmness initially increased during the early stages of storage (Weeks 2–4), followed by a decline in later weeks across all packaging materials (Figure 2D–F). The early increase in fruit firmness was likely caused by initial surface dehydration [63]; however, the subsequent loss of firmness is associated with overall water loss, as has been reported for Ortanique mandarins [64] and Fortune mandarins [65]. The greatest change in firmness was observed at 25 °C. At lower temperatures (8 °C and 4 °C), firmness was maintained more consistently throughout the storage period. At Week 8, fruit stored in punnets had the highest numerical firmness values at all three temperatures. At RT, fruit stored in punnets were significantly firmer than fruit stored in either cardboard clamshells or net bags, whereas at 4 °C, fruit stored in punnets were significantly firmer than fruit stored in net bags. Perforated punnets were slightly more effective at retaining firmness than cardboard clamshells and net bags during the early storage period (approximately Weeks 2–4). No significant packaging-related differences were detected at 8 °C.

### 3.3. Colour, Visual Storage Disorders, Fungal Growth and Marketability

During this eight-week trial, the greatest change in skin colour (as assessed by ΔE* values) was associated with storage temperature, with the greatest change occurring at RT and the least at 4 °C (Figure 3A,C), although the difference between fruit stored at 8 °C and 4 °C was small. Packaging had a smaller effect on ΔE* values. At room temperature, there were significant differences in ΔE* among the three packaging treatments, whereas at the other two temperatures, only fruit stored in net bags differed significantly from fruit stored in the other two packaging types. Nonetheless, there was a non-significant trend for punnets to preserve skin colour better than cardboard packaging (Figure 4).

The major symptoms of chilling injury in citrus species include pitting, a superficial scald-like appearance of the peel, skin browning and collapse of affected areas, resulting in depressions in the fruit surface [66,67,68]. These symptoms not only reduce visual appeal to consumers but may also create sites for secondary pathogen infection, promoting spoilage and facilitating pathogen growth and sporulation that can spread to nearby fruit.

Table 1 presents the visual assessments of fruit subjected to each storage temperature × packaging treatment. No symptoms of chilling injury or visible fungal growth were observed in fruit stored at RT or at 8 °C in any packaging type. At 4 °C, chilling injury occurred by Week 3, and fungal growth appeared by Week 4 in fruit stored in net bags and cardboard clamshells. The development of these symptoms was delayed by one week in fruit stored in punnets compared with the other packaging types. The onset and severity of chilling injury vary among citrus species and cultivars, because sensitivity to low temperature is genotype-dependent and is also influenced by fruit maturity and the duration of cold exposure [45,46,66,67,68]. Therefore, the chilling response observed in ‘Red Champagne’ finger lime at 4 °C may differ in timing and severity from that reported for other citrus cultivars. The maximum chilling-injury severity reached a score of 2, corresponding to moderate injury, in all packaging types. Shrivelling first appeared at RT during Week 1 and progressively increased in severity; fruit in all packaging treatments at RT became unsaleable after approximately 10 days because of pronounced shrivelling and desiccation. Although the fruit were no longer commercially marketable, measurements were continued to investigate the progression of postharvest deterioration and to compare storage trajectories among temperature treatments. Shrivelling was not observed at the other two temperatures. However, approximately 60% of fruit stored in cardboard clamshells and net bags were classified as unsaleable at Week 8 because of loss of freshness, whereas approximately 80% of fruit stored in punnets remained saleable. Therefore, punnets outperformed cardboard clamshells and net bags in terms of fruit preservation. Comparable effects have been reported in other citrus fruits. Baswal et al. found that pinhole-perforated polypropylene packaging used during refrigerated storage of ‘Kinnow’ mandarins significantly reduced weight loss, spoilage, and firmness loss, while maintaining several quality attributes compared with unpackaged fruit [69]. Similar benefits of film-based packaging have also been reported for mandarins and tangelos during postharvest storage [70,71]. The poorer performance of the cardboard clamshells may be related to the ability of cardboard to absorb moisture under humid conditions, which may facilitate greater moisture exchange between the fruit and the surrounding environment [72,73].

### 3.4. Total Phenolic Content

Since phenolic compounds contribute to antioxidant activity and nutritional value, total phenolic content is considered an important indicator of citrus fruit quality. Finger lime contains moderately high levels of phenolic compounds in both the pulp and peel [66]. In the present study, total phenolic content declined progressively from Weeks 2 to 6, with the decline being more pronounced at higher storage temperatures (Figure 5A–C). Fruit stored at 4 °C generally maintained the highest phenolic content, particularly in punnets, whereas fruit stored at room temperature, especially in net bags, showed the lowest values. Across temperatures, punnets generally retained higher phenolic levels than cardboard clamshells or net bags.

The greater retention of phenolic compounds at lower temperatures may be associated with slower metabolic and oxidative reactions during storage, thereby reducing the degradation of these compounds. Conversely, higher temperatures can accelerate oxidative processes and contribute to more rapid phenolic loss. This interpretation is consistent with previous studies reporting greater phenolic degradation at higher storage temperatures [74,75,76] and improved retention of phenolic compounds in citrus products stored at lower temperatures of 6–10 °C during extended storage [31,77,78].

### 3.5. Flavonoid Content

Flavonoids contribute to the antioxidant activity and nutritional quality of citrus fruit and have been associated with the prevention of chronic diseases [79]. Finger lime is a rich source of flavonoids in both the peel and pulp [80]. In this study, the greatest reduction in flavonoid content occurred between Weeks 4 and 6 and became more pronounced with increasing storage temperature (Figure 5D–F). Between Weeks 2 and 6, total flavonoid content decreased by approximately 43–59% at RT and 28–36% at 8 °C, compared with only 14–15% at 4 °C. Fruit stored in punnets retained significantly higher flavonoid content than the other packaging treatments at each temperature, possibly due to the more humid microenvironment within the package, which reduces moisture loss and may slow the deterioration of bioactive compounds during storage [76]. The greater retention of flavonoids at lower temperatures is consistent with previous studies in citrus showing that reduced storage temperatures can slow flavonoid loss [22,81].

### 3.6. Antioxidant Capacity

Finger lime has relatively high antioxidant capacity, which is associated with its phenolic and flavonoid contents [80]. Antioxidant capacity remained relatively stable between Weeks 2 and 4 but declined markedly between Weeks 4 and 6, with the smallest reductions observed at 4 °C (Figure 5G–I). Fruit stored in punnets generally maintained higher antioxidant capacity than fruit stored in net bags. This may be attributed to slower respiration, transpiration and enzyme-mediated metabolic reactions at lower temperatures, which can reduce water loss, delay senescence and slow the degradation of bioactive compounds [23,24,30,58]. These findings are consistent with studies on ‘Afourer’ mandarins, orange juice and lemons reporting declines in antioxidant capacity during storage [55,82,83]. However, responses can vary among citrus species and cultivars; increases in phenolic content and antioxidant activity have been reported in ‘Budd Blood’ orange [31], while genotype-dependent responses have been observed among orange cultivars [78]. Such differences may reflect cultivar-specific metabolism, storage temperature, storage duration, and experimental conditions. It is also acknowledged that the use of three replicate packages per treatment limits the broader generalisation of these findings.

## 4. Conclusions

Storage temperature and packaging type both affected the quality attributes and visual marketability of finger lime. Fruit stored at room temperature showed the greatest weight loss, the most pronounced colour change and the largest decline in bioactive compounds. In contrast, storage at 4 °C better preserved firmness and colour and generally maintained higher levels of bioactive compounds during the period when all temperatures could be compared. However, this advantage was offset by the development of chilling injury and visible fungal growth. At 8 °C, no chilling injury, visible fungal growth or shrivelling was observed, but approximately 60% of fruit in cardboard clamshells and net bags were classified as unsaleable at the end of the experiment because of loss of freshness, whereas approximately 80% of fruit in punnets remained saleable. Therefore, under the conditions of this study, storage at 8 °C in rPET punnets provided the best balance between quality retention and marketability; however, as the study used a single cultivar from one harvest season, further validation across cultivars, seasons and growing conditions is warranted.

The present study measured total phenolic content and monitored visible fungal growth as quality indicators; however, individual phenolic profiling and microbiological isolation, identification and quantification were beyond the scope of this packaging–temperature study and should be considered in future research. Future studies could evaluate edible coatings or natural anti-fungal treatments to further reduce chilling injury and fungal decay.

## Figures and Tables

**Figure 1:**
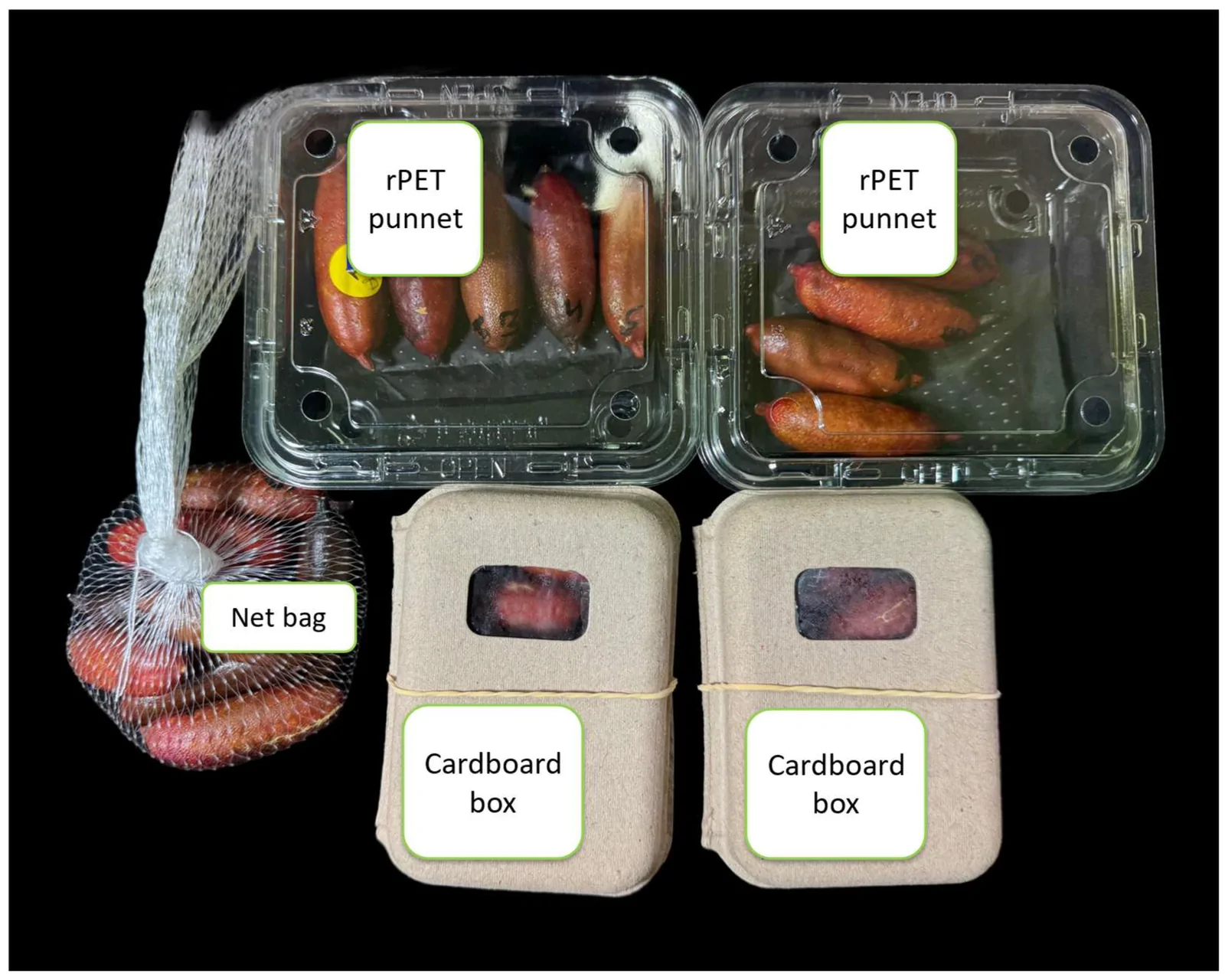
Packaging materials used to evaluate the preservation of finger limes during storage: net bags, rPET punnets and moulded cardboard clamshells.

**Figure 2:**
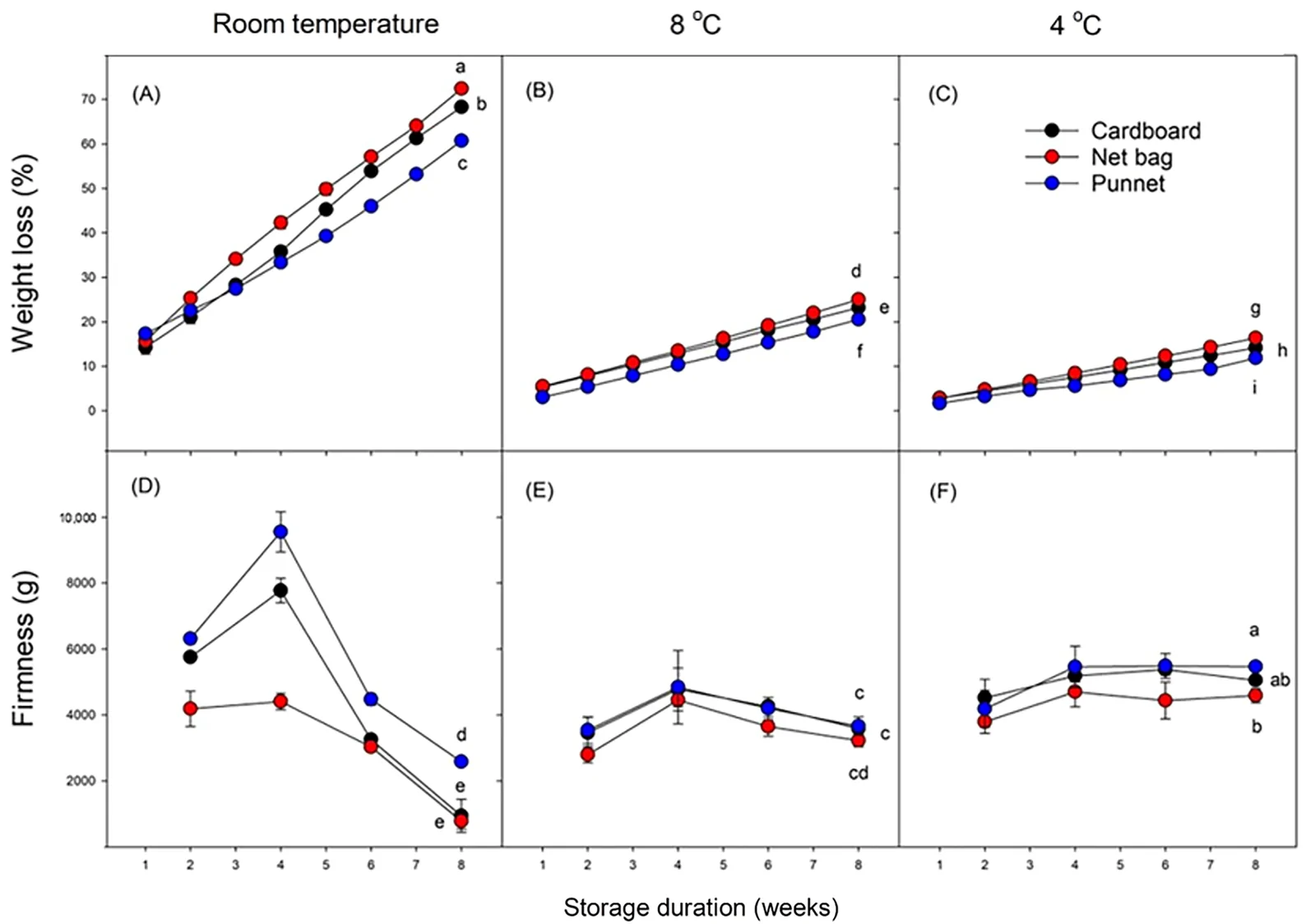
Effect of storage temperature (room temperature, 8 °C and 4 °C) on fruit weight loss (**A**–**C**) and firmness (**D**–**F**) of finger limes stored in different packaging types: cardboard clamshells, punnets and net bags. Data are shown as means ± SE (*n* = 3) over eight weeks of storage. Where error bars are not visible, they are smaller than the symbols. For each parameter, means for all nine treatment combinations at Week 8 annotated with the same letter are not significantly different according to Fisher’s LSD test at *p* = 0.05.

**Figure 3:**
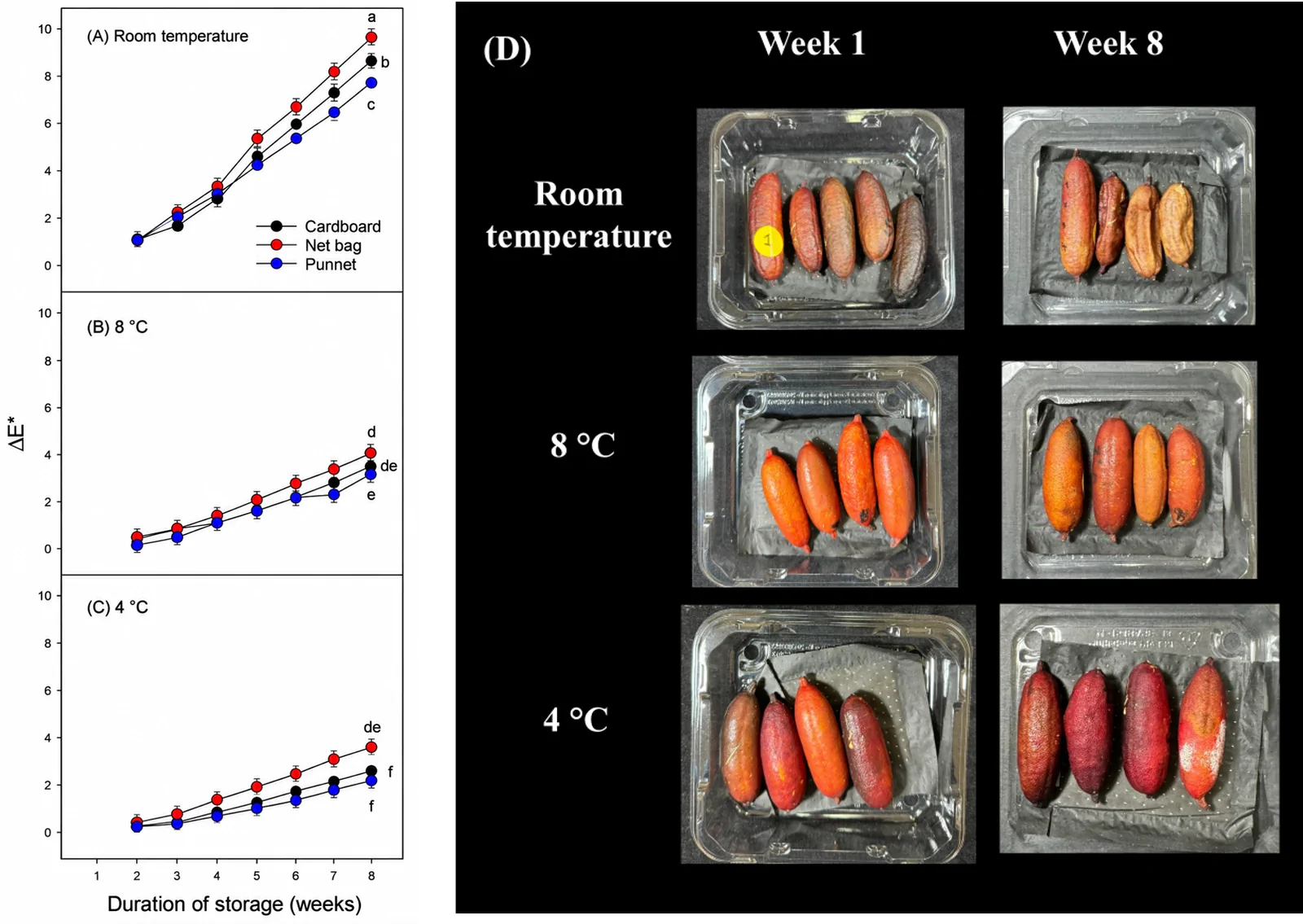
Changes in colour and overall appearance of finger lime fruit during eight weeks of storage. Changes in ΔE* values of finger lime fruit stored at (**A**) room temperature, (**B**) 8 °C and (**C**) 4 °C in different packaging types: cardboard clamshells (●), punnets (●) or net bags (●). Data are shown as means ± SE (*n* = 3). Where error bars are not visible, they are smaller than the symbols. Means for all nine treatment combinations at Week 8 annotated with the same letter are not significantly different according to Fisher’s LSD test at *p* = 0.05. (**D**) Images of finger limes stored in punnets at room temperature, 8 °C and 4 °C after one and eight weeks of storage, showing changes in colour and overall appearance.

**Figure 4:**
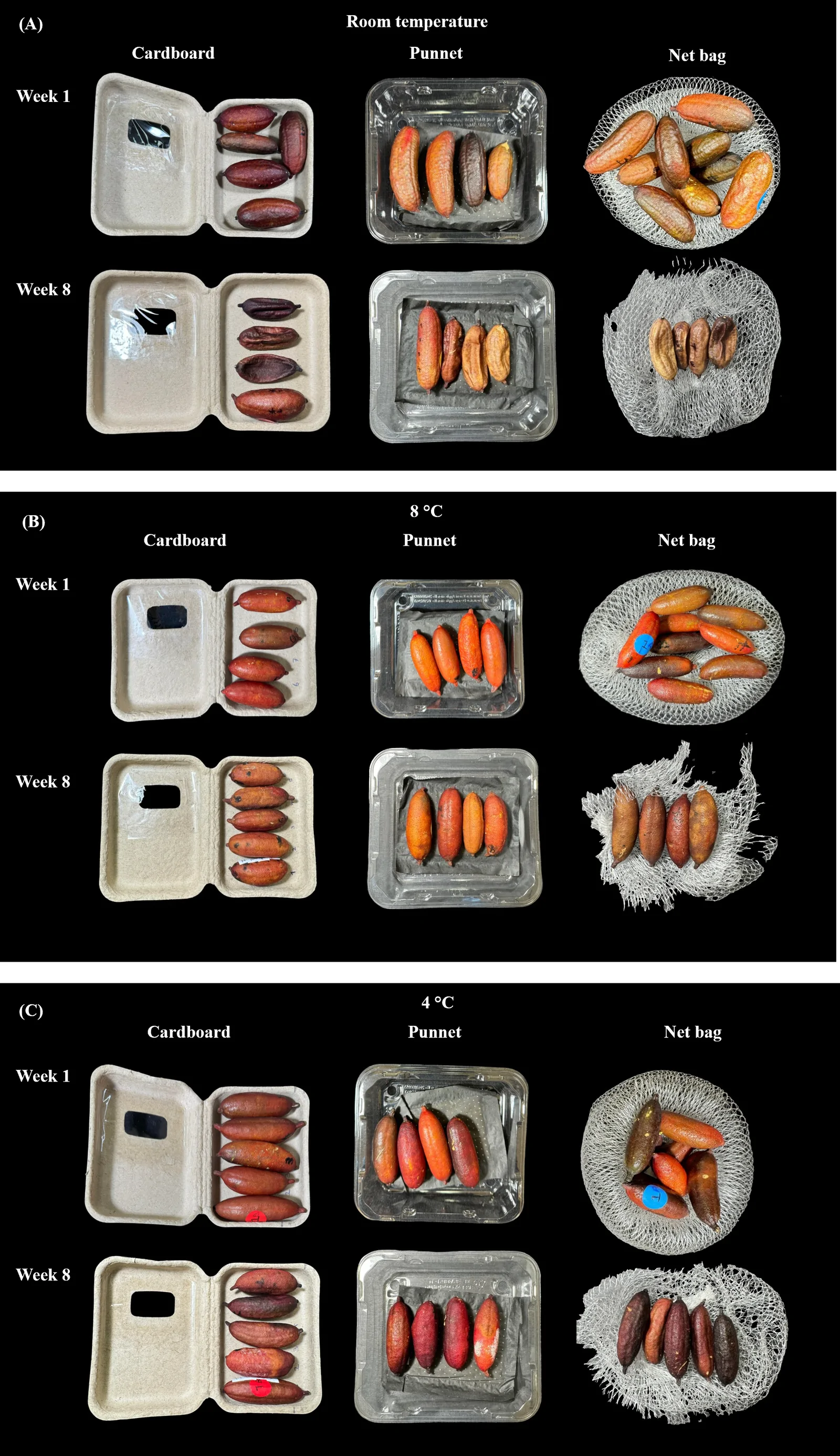
Finger limes stored in different packaging types (cardboard clamshells, punnets and net bags) at (**A**) room temperature, (**B**) 8 °C and (**C**) 4 °C, showing changes in appearance between Week 1 and Week 8 of storage.

**Figure 5:**
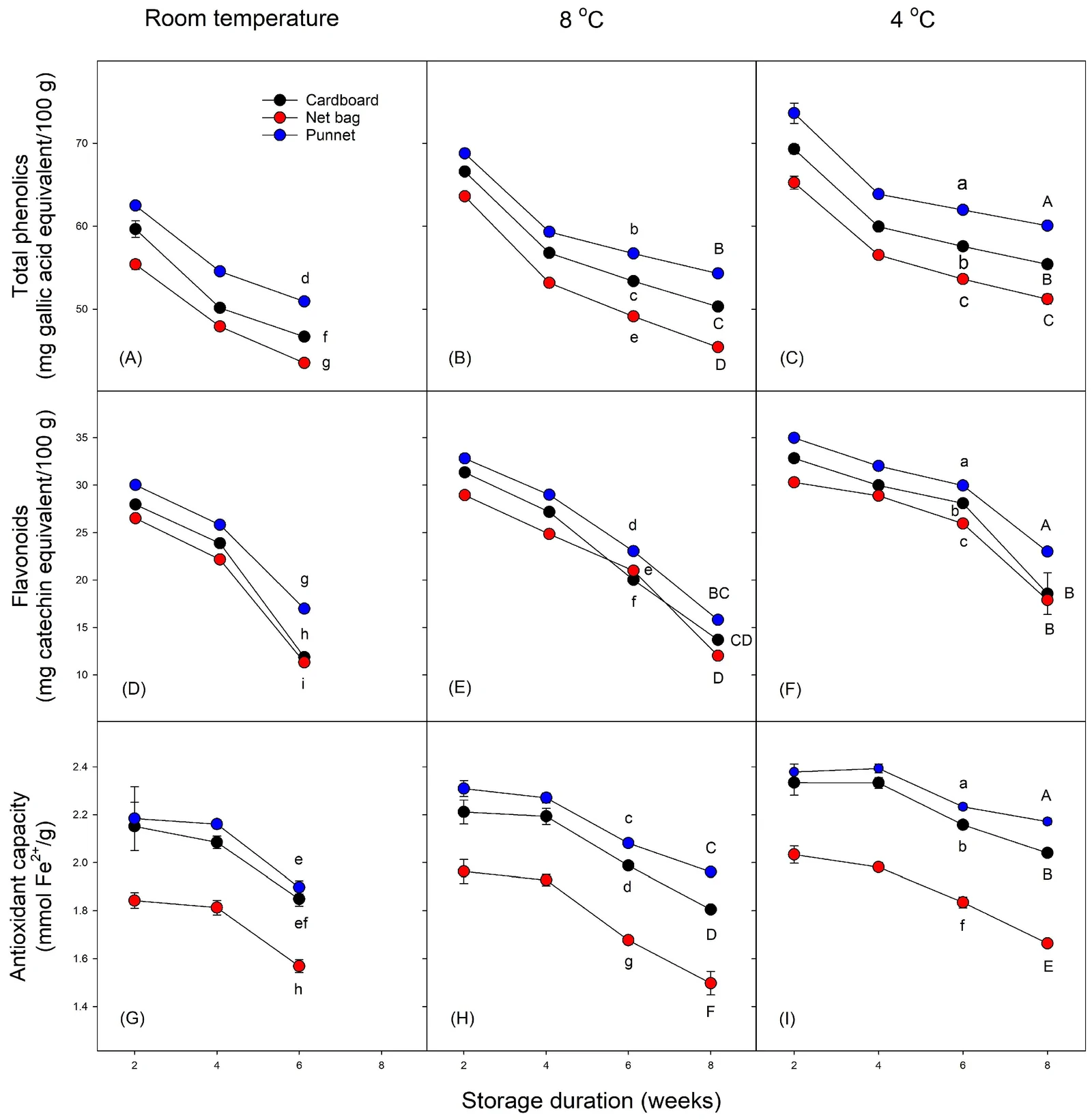
Changes in total phenolic content (**A**–**C**), flavonoid content (**D**–**F**) and antioxidant capacity (**G**–**I**) of finger lime fruit stored in cardboard clamshells (●), punnets (●) or net bags (●) during eight weeks of storage. Data are shown as means ± SE (*n* = 3). Where error bars are not visible, they are smaller than the size of the symbols. For each parameter, means for all nine treatment combinations from the assessment at Week 6 or Week 8 annotated with the same letter (lowercase letters for Week 6 and uppercase letters for Week 8) are not significantly different according to Fisher’s LSD test at *p* = 0.05. Fruit stored at room temperature were completely dry by Week 8; therefore, insufficient pulp/juice was available to measure total phenolic content, antioxidant activity and flavonoid content.

**Table 1:** Effects of storage temperature on the onset and severity of visible storage disorders and marketability of finger lime fruit.

Symptom	RT/Cardboard	RT/Punnet	RT/Net Bag	8 °C/Cardboard	8 °C/Punnet	8 °C/Net Bag	4 °C/Cardboard	4 °C/Punnet	4 °C/Net Bag
First occurrence of chilling injury	Not observed	Not observed	Not observed	Not observed	Not observed	Not observed	Week 3	Week 4	Week 3
Maximum chilling injury severity (0–3)	0	0	0	0	0	0	2, moderate	2, moderate	2, moderate
First week of visible fungal growth	Not observed	Not observed	Not observed	Not observed	Not observed	Not observed	Week 4	Week 5	Week 4
First appearance of shrivelling	Week 1	Week 2	Week 1	Not observed	Not observed	Not observed	Not observed	Not observed	Not observed
Final marketability at Week 8	100% unsaleable	100% unsaleable	100% unsaleable	~60% unsaleable	~20% unsaleable	~60% unsaleable	~20% unsaleable	~20% unsaleable	~20% unsaleable
Main reason for reduced marketability	Pronounced shrivelling and desiccation	Pronounced shrivelling and desiccation	Pronounced shrivelling and desiccation	Loss of freshness	Fruit remained saleable	Loss of freshness	Moderate chilling injury and visible fungal growth	Moderate chilling injury and visible fungal growth	Moderate chilling injury and visible fungal growth

## Data Availability

The original contributions presented in this study are included in the article and Supplementary Materials. Further inquiries can be directed to the corresponding author.

## Supplementary Materials

The following supporting information can be downloaded at https://www.mdpi.com/article/10.3390/foods15193476/s1. Table S1. Statistical analysis for weight loss; Table S2. Statistical analysis for firmness; Table S3. Statistical analysis for colour; Table S4. Statistical analysis for total phenolic content; Table S5. Statistical analysis for total flavonoid content; and Table S6. Statistical analysis for total antioxidant capacity.

## Abbreviations

The following abbreviations are used in this manuscript:

rPET	recycled polyethylene terephthalate
RT	room temperature
FRAP	ferric reducing antioxidant power
FW	fresh weight

## References

[1] Mabberley D.J. (2022). A classification for edible citrus: An update, with a note on *Murraya* (Rutaceae). Telopea.

[2] Johnson J.B., Hungerford N.L., Sultanbawa Y., Netzel M.E. (2025). Unlocking the sublime: A review of native Australian citrus species. Foods.

[3] Glover R., Taylor T., Redman M., Trainer P. (2021). Australian Native Finger Lime RD&E Plan (2023–2028). https://agrifutures.com.au/product/australian-native-finger-lime-rde-plan-2023-2028/.

[4] Qi Y., Liu H., Agar O.T., Imran A., de Souza T.S.P., Barrow C., Dunshea F., Suleria H.A.R. (2024). Phytochemicals in finger lime and their potential health benefits: A review. Food Rev. Int..

[5] Aznar R., Rodríguez-Pérez C., Rai D.K. (2022). Comprehensive characterization and quantification of antioxidant compounds in finger lime (*Citrus australasica* L.) by HPLC-QTof-MS and UPLC-MS/MS. Appl. Sci..

[6] Hardy S., Wilk P., Viola J., Rennie S. (2010). Growing Australian Native Finger Limes. https://www.dpi.nsw.gov.au/__data/assets/pdf_file/0016/320272/growing-australian-native-finger-limes.pdf.

[7] Nakandala U., Furtado A., Masouleh A.K., Smith M.W., Williams D.C., Henry R.J. (2024). The genome of *Citrus australasica* reveals disease resistance and other species specific genes. BMC Plant Biol..

[8] Lu X., Zhao C., Shi H., Liao Y., Xu F., Du H., Xiao H., Zheng J. (2023). Nutrients and bioactives in citrus fruits: Different citrus varieties, fruit parts, and growth stages. Crit. Rev. Food Sci. Nutr..

[9] Suri S., Singh A., Nema P.K., Singh Purewal S., Punia Bangar S., Kaur P. (2023). Bioactive compounds in citrus fruits: Extraction and identification. Recent Advances in Citrus Fruits.

[10] Samanta S., Banerjee J., Ahmed R., Dash S.K., Singh Purewal S., Punia Bangar S., Kaur P. (2023). Potential benefits of bioactive functional components of citrus fruits for health promotion and disease prevention. Recent Advances in Citrus Fruits.

[11] Burnett C.L., Bergfeld W.F., Belsito D.V., Hill R.A., Klaassen C.D., Liebler D.C., Marks J.J.G., Shank R.C., Slaga T.J., Snyder P.W. (2021). Safety assessment of *Citrus* peel-derived ingredients as used in cosmetics. Int. J. Toxicol..

[12] Smeriglio A., Cornara L., Denaro M., Barreca D., Burlando B., Xiao J., Trombetta D. (2019). Antioxidant and cytoprotective activities of an ancient Mediterranean citrus (*Citrus lumia* Risso) albedo extract: Microscopic observations and polyphenol characterization. Food Chem..

[13] Mazzotti F., Bartella L., Talarico I.R., Napoli A., Di Donna L. (2021). High-throughput determination of flavanone-O-glycosides in citrus beverages by paper spray tandem mass spectrometry. Food Chem..

[14] Zhang H., Tian G., Zhao C., Han Y., DiMarco-Crook C., Lu C., Bao Y., Li C., Xiao H., Zheng J. (2019). Characterization of polymethoxyflavone demethylation during drying processes of citrus peels. Food Funct..

[15] Peng Z., Zhang H., Li W., Yuan Z., Xie Z., Zhang H., Cheng Y., Chen J., Xu J. (2021). Comparative profiling and natural variation of polymethoxylated flavones in various citrus germplasms. Food Chem..

[16] Saini R.K., Ranjit A., Sharma K., Prasad P., Shang X., Gowda K.G.M., Keum Y.-S. (2022). Bioactive compounds of citrus fruits: A review of composition and health benefits of carotenoids, flavonoids, limonoids, and terpenes. Antioxidants.

[17] Dhuique-Mayer C., Servent A. (2025). An overview of the nutritional quality and health benefits linked to the world diversity of citrus fruits/juices. J. Food Sci..

[18] Delort E., Yuan Y.-M., Rodrigues S., de Oliveira Silva E., de Brito E.S. (2018). Finger lime/The Australian Caviar—*Citrus australasica*. Exotic Fruits.

[19] Adhikari B., Dutt M., Vashisth T. (2021). Comparative phytochemical analysis of the fruits of four Florida-grown finger lime (*Citrus australasica*) selections. LWT.

[20] Sdiri S., Salvador A., Farhat I., Navarro P., Besada C., Hayat K. (2014). Influence of postharvest handling on antioxidant compounds of citrus fruits. Citrus: Molecular Phylogeny, Antioxidant Properties and Medicinal Uses.

[21] Palou L., Valencia-Chamorro S.A., Pérez-Gago M.B. (2015). Antifungal edible coatings for fresh citrus fruit: A review. Coatings.

[22] Baltazari A., Mtui H.D., Mwatawala M.W., Chove L.M., Msogoya T., Samwel J., Subramanian J. (2020). Effects of storage conditions, storage duration and post-harvest treatments on nutritional and sensory quality of orange (*Citrus sinensis* (L) Osbeck) fruits. Int. J. Fruit Sci..

[23] Wills R., McGlasson B., Graham D., Joyce D.C. (2007). Postharvest: An Introduction to the Physiology and Handling of Fruit, Vegetables and Ornamentals.

[24] Wills R.B.H., Golding J.B., Wills R.B.H., Golding J.B. (2016). Physiology and biochemistry. Postharvest: An Introduction to the Physiology and Handling of Fruit and Vegetables.

[25] Strano M.C., Altieri G., Allegra M., Di Renzo G.C., Paterna G., Matera A., Genovese F. (2022). Postharvest technologies of fresh citrus fruit: Advances and recent developments for the loss reduction during handling and storage. Horticulturae.

[26] Redacción (2025). The Rise of Finger Lime: From Global Trade Routes to Postharvest Challenges. Postharvest.

[27] Xu S., Dai X., Zhao W., Yang X., Tang R. (2026). Systematic comparison of the effects of different treatments on the shelf-life storage of citrus fruits and screening of preservation measures: A frequency network meta-analysis. Food Chem. X.

[28] El-Otmani M., Ait-Oubahou A., Zacarías L., Yahia E.M. (2011). 21—*Citrus* spp.: Orange, mandarin, tangerine, clementine, grapefruit, pomelo, lemon and lime. Postharvest Biology and Technology of Tropical and Subtropical Fruits.

[29] Rey F., Zacarías L., Rodrigo M.J. (2020). Carotenoids, vitamin C, and antioxidant capacity in the peel of mandarin fruit in relation to the susceptibility to chilling injury during postharvest cold storage. Antioxidants.

[30] Malik S., Singh A., Shahi N.C., Bhat M.I., Richa R., Naik B., Rustagi S., Khan J.M., Gupta A.K., Kumar V. (2024). Optimization of pretreatments to enhance quality characteristics and storage period of kinnow (*Citrus reticulata* Blanco) using response surface methodology. Sci. Rep..

[31] Habibi F., Shahid M.A., Spicer R.L., Voiniciuc C., Kim J., Brecht J.K., Sarkhosh A. (2024). Postharvest storage temperature strategies affect anthocyanin levels, total phenolic content, antioxidant activity, chemical attributes of juice, and physical qualities of blood orange fruit. Food Chem. Adv..

[32] Alemu T.T., Intipunya P., Gebeyo B.A. (2025). A comprehensive review of edible coatings for postharvest management of fruits and vegetables: Enhancing food and nutrition security. Discov. Agric..

[33] Dong H., Tong L., Hou S. (2025). Humidity control in fruits and vegetables packaging using bilayer asymmetric films for reduced postharvest losses. Trends Food Sci. Technol..

[34] Bajwa B.E., Anjum F.M. (2007). Improving storage performance of *Citrus reticulata* Blanco mandarins by controlling some physiological disorders. Int. J. Food Sci. Technol..

[35] Tietel Z., Lewinsohn E., Fallik E., Porat R. (2012). Importance of storage temperatures in maintaining flavor and quality of mandarins. Postharvest Biol. Technol..

[36] Rivera F., Pelayo-Zaldivar C., De LeÓN F.D., Buentello B., Castillo-Rivera M., Pérez-Flores L.J. (2007). Cold-conditioning treatment reduces chilling injury in mexican limes (*Citrus aurantifolia* S.) stored at different temperatures. J. Food Qual..

[37] Eaks I.L., Masias E. (1965). Chemical and physiological changes in lime fruits during and after storage. J. Food Sci..

[38] Ladaniya M.S. (2004). Response of ‘Kagzi’ acid lime to low temperature regimes during storage. J. Food Sci. Technol..

[39] Rural Industries Research and Development Corporation (2014). Focus on Finger Lime. https://agrifutures.com.au/product/focus-on-finger-lime/.

[40] Al Shoffe Y., Johnson L.K. (2024). Opportunities for prediction models to reduce food loss and waste in the postharvest chain of horticultural crops. Sustainability.

[41] Rehal J., Kaur G.J., Bons H.K. (2017). Studies on physico-mechanical properties of *W. Murcott* mandarin. J. Appl. Nat. Sci..

[42] Carter E.C., Schanda J.D., Hirschler R., Jost S., Luo M.R., Melgosa M., Ohno Y., Pointer M.R., Rich D.C., Viénot F. (2018). Technical Report—Colorimetry.

[43] McGuire R.G. (1992). Reporting of objective color measurements. HortScience.

[44] Palma A., D’Aquino S., Vanadia S., Angioni A., Schirra M. (2013). Cold quarantine responses of ‘Tarocco’ oranges to short hot water and thiabendazole postharvest dip treatments. Postharvest Biol. Technol..

[45] Chaudhary P.R., Jayaprakasha G., Porat R., Patil B.S. (2014). Low temperature conditioning reduces chilling injury while maintaining quality and certain bioactive compounds of ‘Star Ruby’ grapefruit. Food Chem..

[46] Habibi F., Ramezanian A., Guillén F., Martínez-Romero D., Serrano M., Valero D. (2020). Susceptibility of blood orange cultivars to chilling injury based on antioxidant system and physiological and biochemical responses at different storage temperatures. Foods.

[47] Liguori G., Greco G., Salsi G., Garofalo G., Gaglio R., Barbera M., Greco C., Orlando S., Fascella G., Mammano M.M. (2024). Effect of the gellan-based edible coating enriched with oregano essential oil on the preservation of the ‘Tardivo di Ciaculli’ mandarin (*Citrus reticulata* Blanco cv. Tardivo di Ciaculli). Front. Sustain. Food Syst..

[48] Gutiérrez-Villamil D.A., Balaguera-López H.E., Álvarez-Herrera J.G. (2023). Brassinosteroids improve postharvest quality, antioxidant compounds, and reduce chilling injury in ‘Arrayana’ mandarin fruits under cold storage. Horticulturae.

[49] Singleton V.L., Rossi J.A. (1965). Colorimetry of total phenolics with phosphomolybdic-phosphotungstic acid reagents. Am. J. Enol. Viticul..

[50] Singleton V.L., Orthofer R., Lamuela-Raventós R.M. (1999). Analysis of total phenols and other oxidation substrates and antioxidants by means of Folin-Ciocalteu reagent. Methods Enzymol..

[51] Blainski A., Lopes G.C., De Mello J.C.P. (2013). Application and analysis of the Folin Ciocalteu method for the determination of the total phenolic content from *Limonium brasiliense* L.. Molecules.

[52] Zhishen J., Mengcheng T., Jianming W. (1999). The determination of flavonoid contents in mulberry and their scavenging effects on superoxide radicals. Food Chem..

[53] Benzie I.F.F., Strain J.J. (1996). The ferric reducing ability of plasma (FRAP) as a measure of “antioxidant power”: The FRAP assay. Anal. Biochem..

[54] Kader A.A. (2013). Postharvest technology of horticultural crops—An overview from farm to fork. Ethiop. J. Appl. Sci. Technol..

[55] Alhassan N., Golding J.B., Wills R.B.H., Bowyer M.C., Pristijono P. (2019). Long term exposure to low ethylene and storage temperatures delays calyx senescence and maintains ‘Afourer’ mandarins and navel oranges quality. Foods.

[56] Błaszczyk J., Bieniasz M., Nawrocki J., Kopeć M., Mierzwa-Hersztek M., Gondek K., Zaleski T., Knaga J., Bogdał S. (2022). The effect of harvest date and storage conditions on the quality of remontant strawberry cultivars grown in a gutter system under covers. Agriculture.

[57] Hasan M.U., Singh Z., Shah H.M.S., Kaur J., Woodward A. (2024). Water loss: A postharvest quality marker in apple storage. Food Bioprocess. Technol..

[58] Habibi F., Shahid M.A., Jacobson T., Voiniciuc C., Brecht J.K., Sarkhosh A. (2025). Postharvest quality and biochemical changes in blood orange fruit exposed to various non-chilling storage temperatures. Horticulturae.

[59] Lufu R., Ambaw A., Opara U.L. (2020). Water loss of fresh fruit: Influencing pre-harvest, harvest and postharvest factors. Sci. Hort..

[60] Singh K.K., Reddy B.S. (2006). Post-harvest physico-mechanical properties of orange peel and fruit. J. Food Eng..

[61] Porat R., Weiss B., Cohen L., Daus A., Goren R., Droby S. (1999). Effects of ethylene and 1-methylcyclopropene on the postharvest qualities of ‘Shamouti’ oranges. Postharvest Biol. Technol..

[62] Ben Yehoshua S., Peretz J., Moran R., Lavie B., Kim J.J. (2001). Reducing the incidence of superficial flavedo necrosis (noxan) of ‘Shamouti’ oranges (*Citrus sinensis*, Osbeck). Postharvest Biol. Technol..

[63] Gidado M.J., Gunny A.A.N., Gopinath S.C.B., Ali A., Wongs-Aree C., Salleh N.H.M. (2024). Challenges of postharvest water loss in fruits: Mechanisms, influencing factors, and effective control strategies—A comprehensive review. J. Agric. Food Res..

[64] Navarro-Tarazaga M.L., Del Río M.A., Krochta J.M., Pérez-Gago M.B. (2008). Fatty acid effect on hydroxypropyl methylcellulose-beeswax edible film properties and postharvest quality of coated ‘Ortanique’ mandarins. J. Agric. Food Chem..

[65] Perez-Gago M.B., Rojas C., DelRio M.A. (2002). Effect of lipid type and amount of edible hydroxypropyl methylcellulose-lipid composite coatings used to protect postharvest quality of mandarins cv. Fortune. J. Food Sci..

[66] Eaks I.L. (1960). Physiological studies of chilling injury in citrus fruits. Plant Physiol..

[67] Cohen E., Shapiro B., Shalom Y., Klein J.D. (1994). Water loss: A nondestructive indicator of enhanced cell membrane permeability of chilling-injured citrus fruit. J. Am. Soc. Hort. Sci..

[68] Lafuente M.T., Zacarias L., Sala J.M., SÃ¡nchez-Ballesta M.T., Gosalbes M.J., Marcos J., González-Candelas L., Lluch Y., Granell A. (2005). Understanding the basis of chilling injury in citrus fruit. Acta Hortic..

[69] Baswal A.K., Dhaliwal H.S., Singh Z., Mahajan B.V.C. (2020). Influence of types of modified atmospheric packaging (MAP) films on cold-storage life and fruit quality of ‘Kinnow’ mandarin (*Citrus nobilis* Lour X *C. deliciosa* Tenora). Int. J. Fruit Sci..

[70] Mahajan B.V.C., Chahal T.S., Dhillon W.S., Kapoor S. (2018). Postharvest quality maintenance of W. Murcott mandarin using packaging films. Indian J. Agric. Sci..

[71] D’Aquino S., Piga A., Agabbio M., McCollum T.G. (1998). Film wrapping delays ageing of `Minneola’ tangelos under shelf-life conditions. Postharvest Biol. Technol..

[72] Modzelewska I. (2006). Climatic conditions vs. hygrostability and strength properties of corrugated board. Folia For. Pol..

[73] Hung D.V., Nakano Y., Tanaka F., Hamanaka D., Uchino T. (2010). Preserving the strength of corrugated cardboard under high humidity condition using nano-sized mists. Compos. Sci. Technol..

[74] Van de Velde F., Güemes D.R., Piagentini A.M., Pirovani M.E. (2013). Health potential and physicochemical attributes after minimal processing and during refrigerated storage of orange (*Citrus sinensis* L., Osbeck). Int. J. Fruit Sci..

[75] Deng M., Deng Y., Dong L., Ma Y., Liu L., Huang F., Wei Z., Zhang Y., Zhang M., Zhang R. (2018). Effect of storage conditions on phenolic profiles and antioxidant activity of litchi pericarp. Molecules.

[76] Huynh T., Kha C., Nguyen V. (2024). Changes in quality parameters and bioactive components of seedless lime fruit (*Citrus latifolia*) during cold storage. Food Res..

[77] Dey P., Hoque M.M., Islam M.Z., Monalisa K. (2020). Kinetics of antioxidants degradation and quality changes of citron (*Citrus medica*) fruit juice concentrate during storage. J. Microbiol. Biotechnol. Food Sci..

[78] Rapisarda P., Bianco M.L., Pannuzzo P., Timpanaro N. (2008). Effect of cold storage on vitamin C, phenolics and antioxidant activity of five orange genotypes [*Citrus sinensis* (L.) Osbeck]. Postharvest Biol. Technol..

[79] Stachelska M.A., Karpiński P., Kruszewski B. (2025). A comprehensive review of biological properties of flavonoids and their role in the prevention of metabolic, cancer and neurodegenerative diseases. Appl. Sci..

[80] Johnson J.B., Thani P.R., Chen S.-Y., Mani J.S., Netzel M.E., Naiker M. (2024). In vitro bioactivity of Australian finger lime cultivars as an initial evaluation of their nutraceutical potential. Nutraceuticals.

[81] Zhang L., Ling W., Yan Z., Liang Y., Guo C., Ouyang Z., Wang X., Kumaravel K., Ye Q., Zhong B. (2020). Effects of storage conditions and heat treatment on the hesperidin concentration in Newhall navel orange (*Citrus sinensis* Osbeck cv. Newhall) juice. J. Food Comp. Anal..

[82] Klimczak I., Małecka M., Szlachta M., Gliszczyńska-Świgło A. (2007). Effect of storage on the content of polyphenols, vitamin C and the antioxidant activity of orange juices. J. Food Comp. Anal..

[83] Sun Y., Singh Z., Tokala V.Y., Heather B. (2019). Harvest maturity stage and cold storage period influence lemon fruit quality. Sci. Hort..

