# Supplementary material for: Effects of Storage Temperature and Packaging Type on the Postharvest Quality of Finger Lime (Citrus australasica)
Source: Foods. 2026 Sep 28;15(19):3476. doi: 10.3390/foods15193476 (PMC13664404; doi:10.3390/foods15193476)
Supplement: Supplementary file 1 [file foods-15-03476-s001.zip › foods-4577033-supplementary.pdf]

foods

MDPI

Article

Effects of Storage Temperature and Packaging Type on the Postharvest Quality of Finger Lime (*Citrus australasica*)

Hanieh Noeparast, Sunil K. Panchal, Paul Holford, Michelle Donovan-Mak *

School of Science, Faculty of Engineering, Computing and Science, Western Sydney University, Hawkesbury Campus, Richmond, NSW 2753, Australia; 22113433@student.westernsydney.edu.au (H.N.); s.panchal@westernsydney.edu.au (S.K.P.); p.holford@westernsydney.edu.au (P.H.)

* Correspondence: m.mak@westernsydney.edu.au

Supplementary Tables

Table S1. Statistical analysis for weight loss.

Week	Effect	F	*p*
2	Packaging	27.8	0.000003
	Temperature	1534.6	0.0
	Interaction	7.1	0.0013
4	Packaging	72.8	0.0
	Temperature	2730.3	0.0
	Interaction	6.0	0.003
6	Packaging	164.9	0.0
	Temperature	6707.6	0.0
	Interaction	8.9	0.00037
8	Packaging	132.3	0.0
	Temperature	7410.9	0.0
	Interaction	10.3	0.00016

Table S2. Statistical analysis for firmness.

Week	Effect	F	*p*
2	Packaging	6.4	0.0081
	Temperature	20.7	0.00002
	Interaction	1.3	0.31
4	Packaging	10.6	0.0009
	Temperature	17.4	0.00006
	Interaction	5.6	0.004
6	Packaging	9.5	0.0015
	Temperature	22.2	0.000014
	Interaction	1.7	0.21
8	Packaging	15.2	0.00014
	Temperature	175.7	0.0
	Interaction	3.6	0.026

Table S3. Statistical analysis for colour.

Week	Effect	F	*p*
8	Packaging	45.1	0.0
	Temperature	975.4	0.0
	Interaction	2.4	0.086

Table S4. Statistical analysis for total phenolic content.

Week	Effect	F	*p*
2	Packaging	22.9	0.000011
	Temperature	6.1	0.0096
	Interaction	0.09	0.99
4	Packaging	194.3	0.0
	Temperature	60.0	0.0
	Interaction	1.1	0.39
6	Packaging	278.7	0.0
	Temperature	160.6	0.0
	Interaction	1.1	0.40
8	Packaging	232.5	0.0
	Temperature	274.3	0.0
	Interaction	0.4	0.66

Table S5. Statistical analysis for total flavonoid content.

Week	Effect	F	*p*
2	Packaging	616.0	0.0
	Temperature	805.6	0.0
	Interaction	7.3	0.0011
4	Packaging	570.7	0.0
	Temperature	1723.5	0.0
	Interaction	6.1	0.0028
6	Packaging	997.2	0.0
	Temperature	11998.4	0.0
	Interaction	104.2	0.0
8	Packaging	12.9	0.001
	Temperature	65.4	0.000003
	Interaction	0.8	0.46

Table S6. Statistical analysis of total antioxidant capacity.

Week	Effect	F	*p*
2	Packaging	22.9	0.00001
	Temperature	6.1	0.0096
	Interaction	0.1	0.99
4	Packaging	194.3	0.0
	Temperature	60.0	0.0
	Interaction	1.1	0.39
6	Packaging	278.7	0.0
	Temperature	160.6	0.0
	Interaction	1.1	0.40
8	Packaging	40810.9	0.0
	Temperature	122.9	0.0
	Interaction	246.1	0.0
